# Associations Between Early-Pregnancy Vitamin D Status and Postpartum Depressive and Anxiety Symptoms

**DOI:** 10.1097/PSY.0000000000001328

**Published:** 2024-07-03

**Authors:** Desirée Domacassé, Susanne R. de Rooij, Tanja Vrijkotte, Ank de Jonge, Jens Henrichs

**Affiliations:** From the Amsterdam UMC, location Vrije Universiteit Amsterdam, Midwifery Science (de Jonge, Henrichs); Midwifery Academy Amsterdam Groningen, InHolland (de Jonge, Henrichs); Amsterdam Public Health, Mental Health (de Jonge, Henrichs), Amsterdam; Department of Primary and Long-term Care, University Medical Center Groningen, University of Groningen (Domacassé, de Jonge, Henrichs), Groningen; Amsterdam UMC location University of Amsterdam, Epidemiology and Data Science (de Rooij); Amsterdam UMC location University of Amsterdam, Reproduction and Development Research Institute (de Rooij, Vrijkotte); Amsterdam UMC location University of Amsterdam, Amsterdam Public Health Research Institute, Aging and Later Life, Health Behaviors and Chronic Diseases (de Rooij, Vrijkotte); and Amsterdam UMC, Department of Public and Occupational Health, University of Amsterdam (Vrijkotte), Amsterdam, the Netherlands.

**Keywords:** vitamin D, pregnancy, depressive disorders, anxiety disorders, **ABCD** = Amsterdam Born Children and their Development (ABCD) birth cohort, **BMI** = body mass index, **CES-D** = Center for Epidemiologic Studies Depression, **CI(s)** = confidence interval(s), **CVs** = coefficients of variation, **CRP** = C-reactive protein, **EPDS** = Edinburgh Postnatal Depression Scale, **ORs** = odds ratios, **Perined** = Dutch Perinatal Registration, **PPD** = postpartum depression, **RCTs** = randomized controlled trials, **REF** = reference, **RIVM** = National Institute for Public Health and the Environment, **STAI** = State-Trait Anxiety Inventory

## Abstract

**Objective:**

Maternal postpartum depressive and anxiety symptoms are risk factors for subsequent maternal and child mental health problems. Little is known about the potential role of antepartum vitamin D and C-reactive protein (CRP) in the etiology of maternal postpartum affective symptoms. We investigated associations between antepartum vitamin D status and postpartum depressive and anxiety symptoms and whether antepartum CRP mediated these associations.

**Methods:**

In 2483 participants of the Amsterdam Born Children and their Development prospective cohort, maternal serum vitamin D and CRP were measured at a median of 13 weeks’ gestation. Vitamin D status was defined as deficient (≤29.9 nM), insufficient (30–49.9 nM), sufficient (50–79.9 nM), or normal (≥80 nM). Maternal depressive symptoms (Center for Epidemiologic Studies-Depression) and anxiety (State-Trait Anxiety Inventory) were assessed 3 months postpartum.

**Results:**

After adjustments for confounders, vitamin D deficiency was only associated with increased postpartum anxiety symptoms (*B* = 0.17, 95% confidence interval [CI] = 0.03–0.30, *p* = .017) compared to normal vitamin D levels (≥80 nM). In women not taking vitamin D supplementation (*n* = 2303), vitamin D deficiency was associated with increased postpartum depressive and anxiety symptoms (*B* = 0.14, 95% CI = 0.03–0.28, *p* = .045; and *B* = 0.17, 95% CI = 0.03–0.32, *p =* .015). Antepartum CRP did not mediate these links.

**Conclusions:**

We found some evidence that antepartum vitamin D deficiency was associated with increased postpartum affective symptoms, especially in women not taking vitamin D supplementation. Clinical trials should determine whether vitamin D supplementation can reduce the risk for postpartum affective disorders.

## INTRODUCTION

Maternal postpartum depressive symptoms and postpartum anxiety are the most common maternal mental health problems experienced in the perinatal period, affecting between 10% and 20% of mothers according to previous systematic reviews (1,2). Maternal mental health problems during pregnancy and the perinatal period put mothers at a higher risk for developing postpartum depression (PPD) and psychosis as well as long-term mental health problems (1,3–5). Furthermore, infants of mothers with postpartum depressive and anxiety symptoms have a higher risk of developing insecure infant-mother attachment styles, experiencing delays in cognitive, social, and motor skills, and to develop mental health problems in childhood and adolescence (5–7).

Although a large body of research exists concerning PPD, less is known about (the etiology of) its milder forms, i.e., postpartum depressive symptoms, and comorbid conditions, i.e., anxiety (8). Depressive and anxiety symptoms often co-occur postpartum and are both characterized by general distress (8–11). Nevertheless, depression and anxiety differ in their expression. In case of depression, there is a lack of “positive affect,” whereas anxiety is specifically characterized by physiological stress (11). In addition, a previous study suggests that maternal anxiety and depressive symptoms affect infants’ expression of emotions differently (12). Emotional flatness was found in infants whose mothers reported depressive symptoms, and less emotional stability was found among infants of anxious mothers (12).

The development of postpartum depressive and anxiety symptoms often starts during pregnancy and is not only triggered by antepartum psychosocial factors but may also be instigated by (neuro)biological factors, including poor micronutrient status (13,14). Previous research suggests that vitamin D deficiency during pregnancy is involved in the development of antepartum and postpartum depression and depressive symptomatology (13,15–19). More recent research also suggests a link between vitamin D deficiency during pregnancy and peripartum anxiety (20). Research investigating the association between antepartum vitamin D status and peripartum anxiety is, however, sparse, and the few existing studies investigating this association reported conflicting results (20,21).

Nielsen et al. (18) suggested that the association between vitamin D, which is a prohormone and an essential micronutrient, and (postpartum) mental health seems biologically plausible. That is, the large presence of vitamin D receptors and 1α-hydroxylase, i.e., the enzyme responsible for forming active vitamin D, in the human brain suggests that vitamin D may have a substantial role in certain mental processes (22). It is therefore conceivable that a lack of stimulation of vitamin D receptors due to vitamin D deficiency could lead to inadequate functioning of hormonal processes in the human brain normally preventing mood disorders (23,24). However, the concrete mechanism responsible for the association between deficient vitamin D levels and adverse peripartum mental health is unknown. One of the potential explanatory factors concerns the activity of proinflammatory cytokines and neuroinflammation, which have been shown to be involved in (postpartum) depression, as supported by earlier reviews and a meta-analysis based on nonpregnant people (25,26). Moreover, C-reactive protein (CRP) concerns a nonspecific, potential biomarker of inflammation that has been proposed to also mirror inflammatory activity and processes in the brain (27). Indeed, elevated levels of the inflammatory biomarker CRP have been found in patients suffering from mood disorders and related to depressive symptoms in nonpregnant populations (26,28,29). CRP in the peripartum period has also been associated with being at risk of postpartum depression (30,31). Importantly, although in theory, vitamin D and CRP levels might be interdependent, a recent large-scale Mendelian randomization study (*n* = 294. 970) convincingly demonstrated that vitamin D status exerts a causal effect on CRP levels but not vice versa (32). Overall, this earlier research into the interconnectedness between vitamin D levels, CRP, and postpartum depressive symptoms suggests that CRP may constitute an intermediate in the association between early-pregnancy vitamin D status and maternal peripartum affective functioning.

The current study was based on the large-scale Amsterdam Born Children and their Development (ABCD) birth cohort, including a large proportion of women with a non-Western ethnicity, who are often at a higher risk of an insufficient vitamin D status (17,25,33). The aim of this study was to investigate whether deficient antepartum vitamin D status is a risk factor for increased postpartum depressive and/or anxiety symptoms. Moreover, this study examined whether antepartum CRP levels mediated these associations.

## METHODS

### Study Population

Data were collected in the ABCD study, a large-scale prospective population-based cohort study (33). For this population-based cohort, 12,373 pregnant women, who lived in Amsterdam, were approached for participation via their antenatal care provider (midwife, obstetrician, or general practitioners) during their first antenatal care visit between January 2003 and March 2004. Of these women, 4389 women (53%) took part in the biomarker study at a median of 13 weeks’ gestation. Due to the population-based design, no inclusion or exclusion criteria were applied for the recruitment of the ABCD study and its embedded biomarker study. More details about the design and data collection of the ABCD study have previously been described elsewhere (33).

Approval for the ABCD study was given by The Central Committee on Research Involving Human Subjects in the Netherlands, the hospitals’ medical ethics review committees, and the Registration Committee of the Municipality of Amsterdam. Written informed consent was given by all participating mothers (33).

### Measures

Blood samples were collected at a median duration of 13 weeks’ gestation (interquartile range = 12–14) after the first antepartum visit to the midwife using a 9-ml Vacuette (Greiner BV, Alphen aan den Rijn, the Netherlands). These samples were sent to the Regional Laboratory of Health Protection Research in Amsterdam, where 1-mL serum aliquots were obtained through centrifugation (1600*g* for 10 minutes at room temperature) and stored at 80°C (17,33,34).

### Vitamin D

Serum 25(OH)D level (nM) was determined by the National Institute for Public Health and the Environment (RIVM, Bilthoven, the Netherlands) with the use of an enzyme immunoassay method (OCTEIA AC-57F1; IDS Ltd, Boldon, UK). The intra-assay and interassay coefficients of variation (CVs) for serum 25(OH)D were below 8% for low values and below 10% for high values (17). Serum levels below 6 nM and above 544 nM were deemed unreliable and excluded (17).

Maternal vitamin D was expressed as a continuous variable as well as categorized to examine a potential threshold effect. In the absence of a universally accepted standard of vitamin D deficiency (35), the following cutoff points, used in previous Dutch and international research, were established: deficient (≤29.9 nM), insufficient (30–49.9 nM), sufficient (50–79.9 nM), or normal (≥80 nM) (17,36–40). Perinatal vitamin D supplementation was reported retrospectively by the participants at 3 months postpartum (33).

### CRP

A Dade-Behring high-sensitivity assay was used to measure antepartum CRP status (mg/L) at the Medical Laboratories of Dr. Stein and colleagues, Maastricht, the Netherlands. The intra-assay and interassay CVs for CRP were 4.1% and 5.2% for low values, respectively, and 0.9% and 2.6% for high values, respectively. CRP concentrations below 0.25 mg/L and above 100 mg/L were excluded from the analyses and considered either below detection level or unreliable. We excluded participants (*n* = 8) taking medication possibly influencing antepartum CRP levels in line with previous research.

### Depressive and Anxiety Symptoms

Mothers were provided with questionnaires, made available in different languages, at 3 months postpartum to assess affective symptoms (17). The Center for Epidemiologic Studies Depression (CES-D) scale was used to measure depressive symptoms. The CES-D is a validated and reliable tool to detect depressive symptoms in the peripartum period (41,42). A total score is obtained by summing up the ratings of 20 items based on the previous week on a four-point scale (1 = rarely to 4 = all of the time). Depressive symptoms were considered high using established cutoffs (CES-D score ≥16) (41,42). The CES-D was not originally designed for the measurement of peripartum depressive symptomatology. Nevertheless, the CES-D has been demonstrated to be a reliable and valid self-report instrument and to be strongly correlated with the well-established Edinburgh Postnatal Depression Scale (EPDS) (43,44). Additionally, the CES-D has been shown to adequately identify clinically diagnosed peripartum depression in both pregnant and postpartum women, as indicated by previously reported moderate to very high degrees of sensitivity and specificity and a good diagnostic accuracy as demonstrated by receiver operator characteristic curve analyses (43,45,46). In this study, the internal consistency of the CES-D total score was good, *α* = .90 (3).

In order to measure anxiety symptoms, mothers filled out the State-Trait Anxiety Inventory (STAI), a validated and reliable tool (47). Answers on the 20 items scale are summed up based on a four-point scale rating (1 = rarely to 4 = all of the time). A previously used cutoff for determining high anxiety symptoms was applied (STAI score >42) (48). In this study, the internal consistency of STAI score was *α* = .94 (49).

### Covariates

We selected potential constitutional, demographic, pregnancy-related and lifestyle-related covariates and confounders a priori and based on earlier research addressing the association between antenatal vitamin D status and antepartum or postpartum depressive and/or anxiety symptoms (16–19). We included the following potential confounders: maternal age (years), maternal educational level (number of years of education after primary school; low <6 years, medium 6–10 years, high ≥11 years), maternal employment (employed/unemployed), marital status (married or living together/partner), parity (nulliparous/multiparous), maternal ethnicity (Dutch origin/non-Dutch based on country of birth), body mass index (BMI; kg/m^2^), prenatal smoking (yes/no), pregnancy desirability (a constructed variable summing up the four-item Likert scale ratings of four questions to assess the desirability of pregnancy as depicted by Brandenbarg et al. (17) (Cronbach’s *α* = .80). Higher scores indicate lower pregnancy desirability. Data on the covariates/confounders mentioned above were self-reported and collected from the pregnancy questionnaire of the ABCD study as administered at 13 weeks’ gestation (33). Moreover, information on gestational age and birth weight was derived from medical records filled in by obstetricians and midwives and extracted from the Dutch Perinatal Registration (Perined) (33). Information on the month during which the early pregnancy blood samples were taken was classified as a dichotomous variable indicating season of sampling (i.e., summer [May to October] versus winter [November to April]) in line with the approach by Brandenbarg et al. (17).

### Statistical Analyses

Descriptive statistics of the analytical sample were presented by summarizing numerical variables by mean and standard deviation (SD) or median and range (depending on their distribution). Categorical variables were summarized with counts and percentages. Nonresponse analyses were used to assess selection bias by comparing antepartum vitamin D, antepartum CRP, maternal educational level, ethnicity, and birth weight between responders and nonresponders. The participants were compared across classes of low and high affective symptoms for demographic, lifestyle, and pregnancy-specific characteristics using independent *t* tests for continuous variables and chi-square (*χ*^2^) tests for categorical variables.

Subsequently, multiple linear regression analyses were utilized to examine the association between antepartum vitamin D status as a continuous as well as a categorical variable and maternal postpartum depressive and anxiety symptoms as continuous outcome variables. For these regression models, the continuous outcomes were *z*-standardized. For each category of vitamin D status, we calculated an effect size (i.e., Cohen’s *d*). The regression coefficients (*B*) reflect the mean difference in *z*-standardized anxiety or depressive symptoms between the respective vitamin D status category and the group with a normal vitamin D level. In line with the criteria by Cohen (50), *d* values below <0.20 represent small effect sizes; *d* values about 0.5, moderate effect sizes; and *d* values >0.80, large effect sizes. Regression coefficients and 95% confidence intervals (CIs) were reported. Due to the possibility that the determinant or outcomes might be skewed, we visually inspected the distribution of the residuals of the applied multivariable regression analyses to check the normality assumption, in line with current recommendations for large sample sizes (51).

Next, we categorized four groups of mothers with increased depressive and/or anxiety symptoms. We differentiated between high and low affective symptoms in the postpartum period using the established cutoffs to define high levels of postpartum depressive symptoms (CES-D score ≥16) and anxiety symptoms (STAI score >42) (41,42,48). Based on these dichotomizations, four categories of maternal postpartum affective symptoms were established: a) low depressive/anxiety symptoms, b) high depressive symptoms, c) high anxiety symptoms, and d) comorbid high depressive and anxiety symptoms.

Using multinomial logistic regression, we investigated the specific associations between maternal antepartum vitamin D status and the different classes of maternal postpartum affective symptoms. Odds ratios (ORs) with CIs are shown to express the associations between the predictor and dependent variables.

We examined whether maternal antepartum CRP levels mediated the associations between maternal antepartum vitamin D levels and postpartum affective symptoms (expressed as continuous sum scores). First, potential significant correlations between maternal antepartum vitamin D status, CRP levels, and the outcomes were explored. Then, we investigated whether maternal antepartum CRP levels mediated the possible associations between maternal antepartum vitamin D levels and maternal postpartum depressive or anxiety symptoms using Model 4 of the SPSS macro, PROCESS (52).

The PROCESS macro applies the bootstrap method in order to perform mediation models computing the indirect effect of a potential mediator derived from 10,000 resamples and bias-corrected bootstrap CIs (52). This allows taking into account nonnormality and/or asymmetry of indirect effects (52). To be able to show effect sizes based on the fully adjusted mediation models, we reported completely standardized indirect effects (*β*). Indirect effects demonstrate statistical significance when CIs do not include zero.

In addition, we conducted two sensitivity analyses. First, mothers with vitamin D supplementation were excluded as this can positively influence vitamin D levels and might influence the results (17). Second, as ethnic minority status is a risk factor for vitamin D deficiency, the analyses were repeated among exclusively women from Dutch origin to test whether maternal ethnicity influenced the results (17).

Potential covariates/confounders were selected for the main analyses if they changed the estimates of the main determinant by more than 5% (53). Variables retained included maternal age, maternal educational level, ethnicity, employment, marital status, BMI, and parity. The level of significance for all analyses was set at *α* = .05, and statistical analyses were carried out using SPSS, version 26.0 for Windows. As the current study was explorative and in line with statistical recommendations, we did not correct for multiple comparisons (54,55). The coding and datasets generated and analyzed during the current study are not publicly available but are available from the corresponding author upon reasonable request.

## RESULTS

### Sample Characteristics

Figure S1 (Supplemental Digital Content, http://links.lww.com/PSYMED/B32) presents the flowchart of the current study population. For the ABCD study, 12,373 women were enrolled, 8266 responded to the first ABCD study questionnaire, and 4389 women took part in the biomarker study. Of the biomarker study participants, 2483 mothers had complete data on antepartum vitamin D status and antepartum CRP levels. These mothers also reported information on depressive and anxiety symptoms at 3 months postpartum and had complete data on all potential covariates/confounders.

Nonresponse analyses showed that excluded mothers (*n* = 1906) had significantly lower vitamin D levels (mean [*M*; SD] = 48.6 [32.5] nM versus *M* [SD] = 59.9 [29.9] nM, *p* = .012) but higher CRP levels (*M* [SD] = 6.5 [7.3] mg/L versus *M* [SD] = 5.5 [6.5] mg/L, *p* < .001) than included mothers (*n* = 2483). Infants of excluded mothers had a significant lower birth weight (*M* [SD] = 3291 [750] g versus *M* [SD] = 3483 [550] g, *p* < .001). Included participants were more likely to be highly educated (50.0% versus 27.2%, *χ*^2^ = 239.7, *p* < .001) and from Dutch origin (67.7% versus 42.4%, *χ*^2^ = 296.3, *p* < .001) than those excluded.

Table S1, Supplemental Digital Content, http://links.lww.com/PSYMED/B32, compares four categories of mothers based on their postpartum scores on the CES-D and STAI. The largest group contained the mothers with low scores on both of these questionnaires, showing no or few signs of depressive and/or anxiety symptoms (78.8%). The following two categories of mothers had either high depressive or high anxiety symptoms, e.g., 2.4% and 6.0%, respectively. The last category included mothers with comorbid affective symptoms suffering from both depressive and anxiety symptoms (12.8%). No significant differences in age, gestational age, birth weight, and month of sampling were observed between these groups (see Table S1).

Women with low postpartum affective symptoms were most likely to be highly educated (52.5%), employed (85.1%), and nulliparous (54.2%), and to have a partner (91.6%), whereas women with comorbid affective symptoms displayed lower rates for these demographic factors. Women with comorbid affective symptoms were least likely to be of Dutch origin (46.8%) as compared to the other groups.

Moreover, mothers with low postpartum affective symptoms had the highest level of pregnancy desirability (*M* [SD] = 5.21 [1.7]) compared to the groups of mothers with different types of affective symptoms. Mothers experiencing anxiety symptoms or reporting comorbid affective symptoms had the lowest vitamin D levels (e.g., *M* [SD] = 53.7 [27.7] and *M* [SD] = 51.3 [31.5]). Additionally, women with comorbid affective symptoms had the highest rates of antepartum vitamin D deficiency (32.1%) compared to the other groups.

### Vitamin D Status and Depressive and Anxiety Symptoms

Table 1A shows associations between maternal antepartum vitamin D status and *z*-standardized postpartum depressive symptoms using multivariable linear regression analyses. An inspection of the residuals did not suggest violations of normality revealing that these analyses met the assumption of normality. Higher antepartum vitamin D levels were negatively but modestly associated with postpartum depressive symptoms in the unadjusted model 1 (*B* = −0.00, 95% CI = −0.01 to −0.00, *p* < .001). This association was no longer significant after adjusting for antepartum and demographic covariates/confounders (model 2).

**TABLE 1A:** Associations Between Maternal Antepartum Vitamin D Status and Maternal Postpartum Depressive Symptoms (*N* = 2483)

	Maternal Postpartum Depressive Symptoms *z*-Scores		
	*B*	95% CI	*p*
Vitamin D level per nM*^a^*			
Model 1*^b^*: vitamin D level, nM*^a^*	−0.00	−0.01 to −0.00	<.001
Model 2*^b^*: vitamin D level, nM*^a^*	−0.00	−0.00 to 0.00	.185
Vitamin D level status*^c^*			
Model 1*^b^*			
Normal	REF	REF	REF
Sufficient*^d^*	0.07	−0.03 to 0.17	.198
Insufficient*^d^*	0.11	−0.01 to 0.22	.070
Deficient*^d^*	0.43	0.31 to 0.55	<.001
Model 2*^b^*			
Normal	REF	REF	REF
Sufficient*^d^*	0.04	−0.05 to 0.14	.383
Insufficient*^d^*	0.01	−0.11 to 0.0.12	.909
Deficient*^d^*	0.13	−0.01 to 0.26	.063

CI = confidence interval.

*^a^* Vitamin D levels: *B* represents the mean increase in maternal symptoms *z*-scores for each unit increase in the predictor (i.e., per nM increase in vitamin D level).

*^b^* Model 1 is the crude model, and model 2 has been adjusted for maternal age, maternal educational level, ethnicity, employment, marital status, body mass index, and parity.

*^c^* Vitamin D status categories: *B* represents the mean difference in maternal symptoms *z*-scores between the category of interest and the reference category.

*^d^* Vitamin D cutoffs: deficient (≤29.9 nM), insufficient (30–49.9 nM), sufficient (50–79.9 nM), or normal (≥80 nM).

**TABLE 1B:** Associations Between Maternal Antepartum Vitamin D Status and Maternal Postpartum Anxiety Symptoms (*N* = 2483)

	Maternal Postpartum Anxiety Symptoms *z*-Scores		
	*B*	95% CI	*p*
Vitamin D level per nM*^a^*			
Model 1*^b^*: vitamin D level, nM*^a^*	−0.01	−0.01 to −0.00	<.001
Model 2*^b^*: vitamin D level, nM^a^	−0.00	−0.00 to 0.00	.188
Vitamin D level status*^c^*			
Model 1*^b^*			
Normal	REF	REF	REF
Sufficient*^d^*	0.06	−0.04 to 0.16	.222
Insufficient*^d^*	0.08	−0.04 to 0.20	.173
Deficient*^d^*	0.50	0.38 to 0.61	<.001
Model 2*^b^*			
Normal	REF	REF	REF
Sufficient*^d^*	0.03	−0.06 to 0.13	.491
Insufficient*^d^*	−0.03	−0.14 to 0.09	.626
Deficient*^d^*	0.17	0.03 to 0.30	.017

CI = confidence interval.

*^a^* Vitamin D levels: *B* represents the mean increase in maternal symptoms *z*-scores for each unit increase in the predictor (i.e., per nM increase in vitamin D level).

*^b^* Model 1 is the crude model, and model 2 has been adjusted for maternal age, maternal educational level, ethnicity, employment, marital status, body mass index, and parity.

*^c^* Vitamin D status categories: *B* represents the represents the mean difference in maternal symptoms *z*-scores between the category of interest and the reference category.

*^d^* Vitamin D cutoffs: deficient (≤ 29.9 nM), insufficient (30–49.9 nM), sufficient (50–79.9 nM), or normal (≥ 80 nM).

For anxiety symptoms, the observed associations were similar (Table 1B). Higher antepartum vitamin D levels were negatively but weakly associated with *z*-standardized postpartum anxiety symptoms in the unadjusted model (*B* = −0.01, 95% CI = −0.01 to −0.00, *p* < .001). After adjustment for covariates/confounders, this association was no longer significant.

**TABLE 2:** Multinomial Logistic Regression Analysis Investigating the Link Between Maternal Antepartum Vitamin D Status and Maternal Postpartum High Depressive and/or Anxiety Symptoms

	Mothers Without Depressive or Anxiety Symptoms, *n* = 1966 (79.2%)	Mothers With Depressive Symptoms (CES-D Score ≥16)*, n* = 60 (2.4%)		Mothers With Anxiety Symptoms (STAI ≥42), *n* = 149 (6.0%)		Mothers With Depressive and Anxiety Symptoms (CES-D Score ≥16 and STAI ≥42), *n* = 308 (12.4%)	
Determinants		OR*^a^*	95% CI	OR*^a^*	95% CI	OR*^a^*	95% CI
Continuous							
Model 1							
Vitamin D level, nM	REF	0.99	0.99–1.00	0.99***	0.98–1.00	0.99***	0.98–0.99
Model 2*^b^*							
Vitamin D level, nM	REF	1.00	0.99–1.01	1.00	0.99–1.00	1.00	0.99–1.00
Categorical							
Model 1							
Normal	REF	REF	REF	REF	REF	REF	REF
Sufficient		1.16	0.58–2.31	1.34	0.83–2.15	1.10	0.78–1.56
Insufficient		1.03	0.46–2.32	1.40	0.82–2.38	1.22	0.82–1.80
Deficient		1.63	0.74–3.55	2.17**	1.29–3.64	2.87***	2.01–4.11
Model 2*^b^*							
Normal	REF	REF	REF	REF	REF	REF	REF
Sufficient		1.11	0.55–2.23	1.26	0.78–2.03	1.01	0.71–1.45
Insufficient		0.94	0.41–2.15	1.11	0.64–1.94	0.89	0.59–1.35
Deficient		1.43	0.58–3.54	1.16	0.63–2.16	1.30	0.84–2.00

CES-D = Center for Epidemiologic Studies Depression; STAI = State-Trait Anxiety Inventory; OR = odds ratiol CI = confidence interval.

* *p* < .05.

** *p* < .01.

*** *p* < .001.

*^a^* Odds Ratio (OR) represents the strength of the association between antepartum vitamin D status and maternal affective symptoms.

*^b^* Model 2, is adjusted for the confounders maternal age, maternal educational level, ethnicity, employment, marital status, body mass index, and parity.

When analyzing the association between the four classes of antepartum vitamin D status and affective symptoms, unadjusted linear regression analyses revealed that a deficient vitamin D status during pregnancy was associated with higher affective symptoms in the postpartum period (Tables 1A and 1B). After adjustment, antepartum vitamin D deficiency was still significantly but weakly associated with higher *z*-standardized postpartum anxiety symptoms (*B* = 0.17, 95% CI = 0.03–0.30, *p* = .017), but no longer with postpartum depressive symptoms (Tables 1A and 1B).

### Vitamin D Status and High or Comorbid Postpartum Depressive and Anxiety Symptoms

Table 2 presents the results of multinomial logistic regression analyses, investigating the associations of maternal antepartum vitamin D status with maternal postpartum affective symptom status outcome groups. In the unadjusted model, higher maternal antepartum vitamin D levels were associated with a lower risk of having postpartum anxiety symptoms or having both depressive and anxiety symptoms (OR = 0.99, 95% CI = 0.98–1.00, *p* < .001 and OR = 0.99, 95% CI = 0.98–0.99, *p* < .001, respectively). After adjustment, antepartum vitamin D was not significantly associated with maternal affective symptoms.

Concerning the different groups of vitamin D status during pregnancy, only the deficient group had an increased risk of having high postpartum anxiety symptoms and of having comorbid high postpartum depressive and anxiety symptoms in the unadjusted model (OR = 2.17, 95% CI = 1.29–3.64, *p* = .004 and OR = 2.87, 95% CI = 2.01–4.11, *p* < .001, respectively). These associations were attenuated after adjustment and no longer statistically significant.

### Vitamin D, CRP, and Affective Symptoms: Single Mediation Models

In order to assess the associations of antepartum CRP with all antepartum vitamin D levels and maternal postpartum depressive or anxiety symptoms, correlations were analyzed in a first step. Positive and significant correlations were found between maternal depressive symptoms and antepartum CRP (*r* = 0.05, *p* = .015) and maternal anxiety symptoms and antepartum CRP (*r* = 0.05, *p* = .013). Negative and significant correlations were found between antepartum vitamin D levels and maternal depressive symptoms (*r* = −0.11, *p* < .001) and antepartum vitamin D levels and maternal anxiety symptoms (*r* = −0.14, *p* < .001) and antepartum vitamin D levels and antepartum CRP (*r* = −0.06, *p* = .006).

**FIGURE 1:**
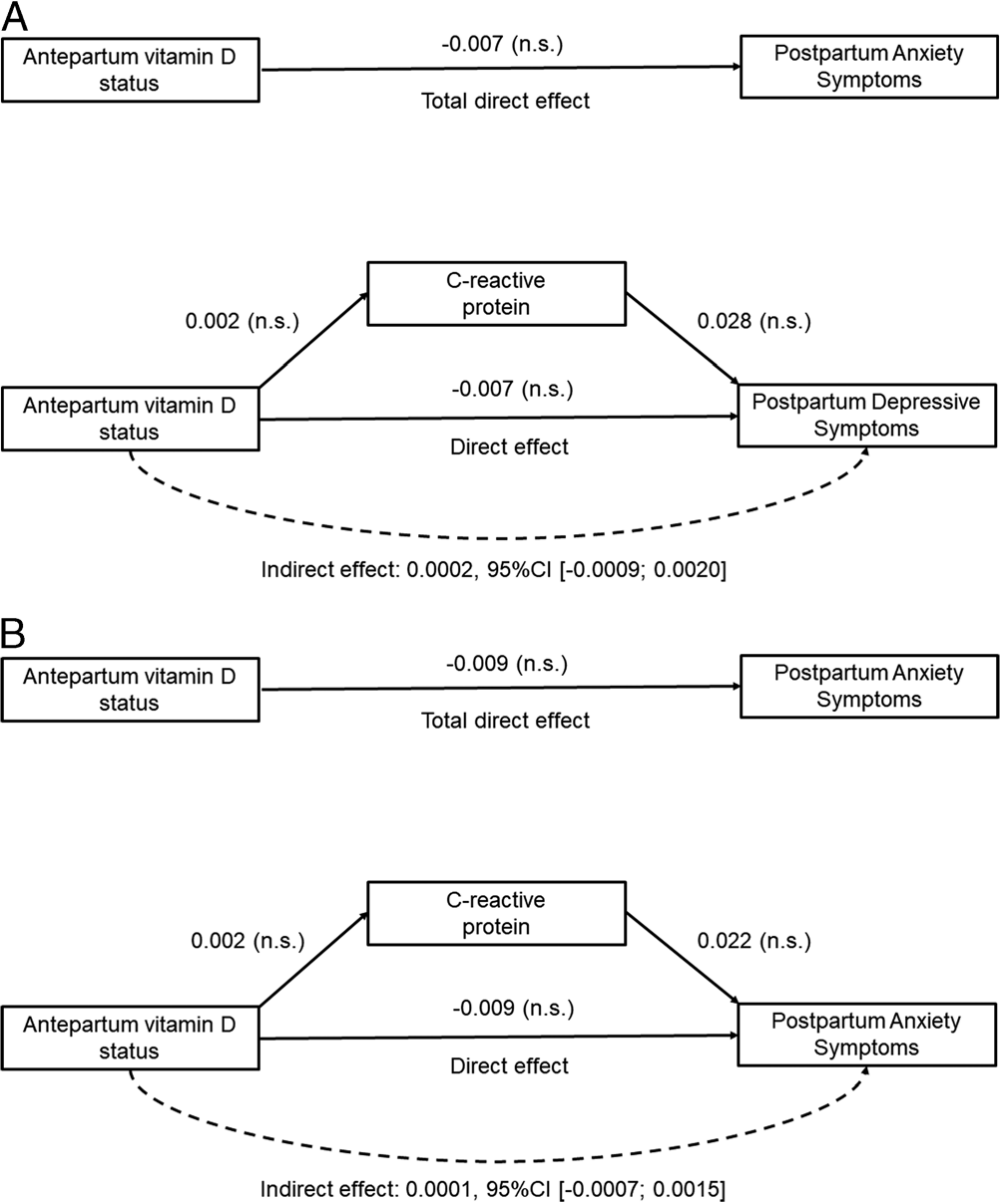
A, Single mediator model of the link between antepartum vitamin D status and postpartum depressive symptoms via antepartum CRP levels after adjustment for covariates/confounders. *Note.* The mediation model testing the association between maternal antepartum vitamin D level and postpartum depressive symptoms via maternal antepartum CRP levels was adjusted for maternal age, maternal educational level, ethnicity, employment, marital status, BMI, and parity. Antepartum CRP was not a significant mediator (*β* = 0.0002, 95% CI = −0.0009 to 0.0020). All other effects tested by this model were also nonsignificant. B, Single mediator model of the link between antepartum vitamin D status and postpartum anxiety via antepartum CRP levels after adjustment for covariates/confounders. *Note.* The mediation model testing the association between maternal antepartum vitamin D level and postpartum anxiety symptoms via maternal antepartum CRP levels was adjusted for maternal age, maternal educational level, ethnicity, employment, marital status, BMI, and parity. Antepartum CRP was not a significant mediator (*β* = 0.0001, 95% CI = −0.0007 to 0.0015). All other effects tested by this model were also nonsignificant. n.s. = nonsignificant. BMI = body mass index; CRP = C-reactive protein.

In a next step, two fully adjusted mediation models tested whether the association between antepartum vitamin D levels and postpartum affective symptoms was mediated via antepartum CRP levels. Antepartum CRP levels were not a significant mediator of the association between antepartum vitamin D levels and postpartum depressive symptoms (*β* = 0.0002, 95% CI = −0.0009 to 0.0020; Figure 1A. In the fully adjusted single mediation model, antepartum vitamin D levels were also not associated with antepartum CRP (*B* = 0.002, 95% CI = −0.008 to 0.011, *p* = .738); and antepartum CRP was not related to postpartum depressive symptoms (*B* = 0.028, 95% CI = −0.015 to 0.070, *p* = .207). Moreover, in this model, antepartum vitamin D levels were not directly associated with postpartum depressive symptoms (*B* = −0.007, 95% CI = −0.017 to 0.003, *p* = .181). As shown in Figure 1B, the fully adjusted single mediation model examining whether antepartum CRP mediated the association between antepartum vitamin D levels and postpartum anxiety revealed the same pattern of results. Again, antepartum CRP levels did not mediate the link between vitamin D levels during pregnancy and postpartum anxiety (*β* = 0.0001, 95% CI = −0.007 to 0.0015). Moreover, antepartum vitamin D levels were not directly associated with postpartum anxiety (*B* = −0.009, 95% CI = −0.023 to 0.004, *p* = .186).

### Sensitivity Analyses

Lastly, sensitivity analyses were performed on two subgroups to examine the associations between antepartum vitamin D status and postpartum depressive or anxiety symptoms among those not taking vitamin D supplementation and when excluding non-Dutch mothers, respectively. Similar results were found for the vitamin D–deficient group among those not using vitamin D supplementation (*n* = 2303). In unadjusted linear regression models, higher levels of antepartum vitamin D were again associated with lower *z*-standardized depressive and anxiety symptoms in the postpartum period (*B* = −0.00, 95% CI = −0.01 to −0.00, *p* < .001; and *B* = −0.00, 95% CI = −0.01 to −0.00, *p* < .001). In the fully adjusted model, these associations were no longer significant. However, after adjustment, vitamin D deficiency was significantly associated with both depressive and anxiety symptoms (*B* = 0.14, 95% CI = 0.03 to 0.28, *p* = .045; and *B* = 0.17, 95% CI = 0.03 to 0.32, *p =* .015, respectively) (Tables S2–S3, Supplemental Digital Content, http://links.lww.com/PSYMED/B32). Based on the sensitivity analyses among women from Dutch origin only (*n* = 1680), no associations were observed between vitamin D status and postpartum depressive and anxiety symptoms after adjustment (Tables S4–S5). The results based on these two additional sensitivity analyses are shown more in detail in Tables S2 to S5.

## DISCUSSION

The current population-based cohort study showed that vitamin D deficiency during pregnancy was associated with increased postpartum anxiety symptoms but not with postpartum depressive symptoms. However, among women not taking vitamin D supplementation during pregnancy, vitamin D deficiency was associated with both increased postpartum depressive and anxiety symptoms. No evidence for a mediating effect by antepartum CRP was found.

Our study extends previous research by addressing postpartum anxiety symptoms, which, despite its distinct expression from postpartum depressive symptoms, remain understudied in the perinatal phase (8,56). Moreover, to the best of our knowledge, this is one of the first studies examining the role of antepartum CRP in the link between antepartum vitamin D levels and postpartum affective symptoms.

Although we found an association between vitamin D deficiency during pregnancy and postpartum affective symptoms, we did not find associations between antepartum vitamin D status with different categories of elevated postpartum affective symptoms. A potential explanation for this latter finding might be a lack of statistical power of the sample when using categorical analyses based on multinomial logistic regression models. Also, after correction for confounders, we observed small effect sizes when analyzing affective symptoms as continuous outcomes. Moreover, for these outcomes, only some of the tested associations were statistically significant. Thus, our findings should be interpreted with caution, as we only found some evidence for an association between early-pregnancy vitamin D status and postpartum affective symptoms.

Although antepartum CRP was significantly but weakly correlated with vitamin D status and postpartum depressive and anxiety symptoms, it was not a mediator of the link between vitamin D status and postpartum affective systems after adjustment for confounders. These findings suggest that other mediators, i.e., possible physiological mechanisms, such as maintenance of calcium homeostasis, may be involved in explaining this link (24).

We conducted a sensitivity analysis excluding those women taking vitamin D supplementation because it can be a strong determinant of vitamin D concentrations. Antepartum vitamin D supplementation might have a protective effect on postpartum mood disorders, including depression and anxiety in the case of vitamin D deficiency due to its lagging effect persisting through the postpartum period (57–61). A biological explanation for the possible protective effect of vitamin D supplementation is implied in the observations that vitamin D metabolites are able to cross the blood-brain barrier and that vitamin D receptors are widespread across brain areas involved in depression (22,62). When excluding participants who took vitamin D supplementation, we found significant associations between antepartum vitamin D deficiency and increased postpartum depressive and anxiety symptoms even after controlling for confounders. This finding thus suggests that women not taking vitamin D supplementation may be more likely to develop both antepartum vitamin D deficiency and postpartum affective symptoms.

Remarkably, among women from Dutch origin, we found no independent association between antepartum vitamin D status and postpartum depressive and/or anxiety symptoms after adjustment for confounders. Within this subgroup of women, an association between vitamin D deficiency and affective symptoms might have gone undetected in our sample due to a possible lack of statistical power (especially when taking the small effect sizes observed for vitamin D into account). Nevertheless, pregnant women from Dutch origin might be at a lower risk of both having vitamin D deficiency and developing postpartum affective symptoms than ethnic minority groups facing more socioeconomic disadvantages, including a lack of personal resources, poorer nutritional status, and inequalities in healthcare consumption (16,63).

The size of the current sample allowed for analyzing whether certain categories of antepartum vitamin D status (e.g., vitamin deficiency [≤29.9 nM]) are specifically associated with postpartum affective symptoms allowing for detection of a potential threshold effect for vitamin D deficiency. This indeed seemed to be the case as vitamin deficiency during pregnancy was associated with postpartum affective symptoms particularly among women not taking vitamin D supplementation. The presence of a threshold effect is in line with earlier studies (23,64).

Several mechanisms may explain the association between antepartum vitamin D levels and postpartum affective functioning. The presence of vitamin D receptors in the central nervous system and human brain, including the changes affecting neurotransmitter activity in case of deficiency, and the protective role vitamin D plays in neuroinflammation support its importance in mood regulation (22,23,58,62). It is important to note that a lack of affect, which is present in depression, is associated with behavior that increases the risk for depressive symptoms including less sunlight exposure by remaining indoors and poor nutrient status due to a decrease in appetite, and therefore, reverse causality might play an important role (58). This implies that depressive symptomatology may also initiate vitamin D deficiency. Longitudinal studies measuring vitamin D levels and affective symptoms repeatedly throughout the perinatal period may address this matter by applying cross-lagged analytic models.

### Strengths and Limitations

Strengths of this study include the biomarker data collected during pregnancy and the measurement of depressive as well as anxiety symptoms 3 months postpartum and the rich information on potential sociodemographic, lifestyle, and pregnancy-specific confounders.

However, the current study also had several limitations. First, one of the limitations of this study is the reliance on self-report of postpartum depressive and anxiety symptoms, which may introduce reporter bias. The Edinburgh Postnatal Depression Scale—Partner Version can be used as an alternative (and addition to self-report) to assess maternal depression through reporting by the partner, which is a validated and reliable measure of depression (65). Second, by including repeated testing of maternal vitamin D status, both antepartum and postpartum, micronutrient status can be evaluated in a more reliable manner allowing the assessment of the (dis-)continuity of vitamin D deficiency. Interestingly, previous research showed that rates of vitamin D deficiency at a population level are relatively stable across the antepartum and postpartum period and that vitamin D levels across pregnancy are strongly correlated (19,66,67). Nevertheless, more research is needed examining in what way timing and (dis-)continuity of vitamin D deficiency in the antepartum and postpartum period are related to postpartum affective symptoms. Third, the design of our study does not fully allow to draw inferences concerning the causality and directionality of effects based on the examined mediation models, as data on the mediator and predictor were collected cross-sectionally. Nevertheless, earlier work supports the theoretical assumptions of the tested mediation models as vitamin D status is predictive of CRP levels and as these two factors have both been shown to be related with depressive symptoms in the peripartum period (18,19,31,32). Fourth, we were not able to correct for history of depression or anxiety in the current study. Future research should incorporate this factor as a confounder in future studies addressing the association between early-pregnancy vitamin D status and postpartum affective symptoms. Fifth, in line with statistical recommendations and due to the explorative nature of the current study, we did not correct for multiple comparisons (54,55). Therefore, more dedicated large-scale studies are required to confirm our findings (55). Finally, nonresponding women had lower antepartum vitamin D concentrations, infants with lower birth weights, and lower levels of education and were less often from Dutch origin than women included in our sample. Possibly, women having such characteristics are more prone to choose for nonparticipation. It is also likely that these women are at a higher risk of postpartum affective symptoms due to more peripartum and sociodemographic adversities.

### Implications for Future Research and Conclusions

Even though this study does not conclusively show that antepartum vitamin D status affects postpartum mental health outcomes, improving peripartum vitamin D levels might provide a simple, safe, and cost-effective approach to mood disorder prevention (58,65). Randomized controlled trials (RCTs) in the peripartum period are needed to shed more light on the potential beneficial effects of vitamin D supplementation on maternal peripartum mental health. These RCTs can also address whether the link between antepartum vitamin D levels and postpartum mental health is causal.

Given the incidence of postpartum anxiety and the risks involved when not identifying women with these symptoms, we suggest further research to focus on anxiety as a separate postpartum mental health problem. For such research, we also recommend measuring postpartum affective symptoms 6 months and a year after giving birth to be able to detect depressive and/or anxiety symptoms, which may develop in a later stage of motherhood.

In conclusion, this study only found some support for a threshold effect for vitamin D deficiency as a risk factor for developing postpartum depressive and/or anxiety symptoms. Antepartum CRP did not appear to mediate this association in this study. Further research is needed to determine whether antepartum vitamin D deficiency poses a risk for developing postpartum depressive and/or anxiety symptoms.
